# Autologous Stem Cell Transplantation in Elderly Patients with Multiple Myeloma: Past, Present, and Future

**DOI:** 10.1155/2014/394792

**Published:** 2014-02-20

**Authors:** Shuji Ozaki, Kazuyuki Shimizu

**Affiliations:** ^1^Department of Hematology, Tokushima Prefectural Central Hospital, 1-10-3 Kuramoto, Tokushima 770-8539, Japan; ^2^Department of Hematology, Tokai Central Hospital, 4-6-2 Sohara-higashijima, Kakamigahara 504-8601, Japan

## Abstract

High-dose melphalan (200 mg/m^2^) as conditioning regimen followed by autologous stem cell transplantation (ASCT) rescue has been established as a standard treatment for patients with multiple myeloma (MM) younger than 65 years of age. However, the role of ASCT in elderly patients older than 65 years remains controversial in the era of novel agents such as thalidomide, bortezomib, and lenalidomide. The efficacy and feasibility of ASCT have been shown in elderly patients by reducing the dose of melphalan to 100–140 mg/m^2^. Although the clinical benefit of reduced-intensity ASCT in elderly patients has not been clearly established in comparison with that of novel agent-based induction therapy, recent studies have demonstrated that sequential strategies of novel agent-based induction therapy and reduced-intensity ASCT followed by consolidation/maintenance with novel agents translate into better outcome in the management of elderly patients. Thus, ASCT could also be a mainstay in the initial treatment of elderly MM patients, and its indication should be evaluated based on performance status and the presence of complications and/or comorbidities of each elderly patient with MM.

## 1. Introduction

Multiple myeloma (MM) is a plasma cell malignancy characterized by the production of monoclonal immunoglobulin and the related organ damages such as hypercalcemia, renal insufficiency, anemia, and lytic bone lesions (CRAB) [1, 2]. Most patients are elderly, aged 65 years or older, and the incidence of MM is increasing according to the aging of general population and an associated increase in life expectancy.

In the 1960s, melphalan + prednisone (MP) therapy was introduced for the treatment of MM, which extended the median survival from approximately 1.5 years to 2 years [3]. Since the late 1990s, high-dose melphalan therapy (200 mg/m^2^) followed by autologous stem cell transplantation (ASCT) has been applied after induction therapy with vincristine + adriamycin + dexamethasone (VAD) in patients younger than 65 years of age, which resulted in the further improvement of survival to 5 years [4, 5]. Consequently, induction therapy + ASCT has been regarded as a standard therapy for younger patients with good health condition, and MP therapy was regarded as a standard of care for elderly patients of 65 years of age or older.

In the early phase of the 21st century, novel agents such as thalidomide, bortezomib, and lenalidomide have entered into clinical practice and become key drugs in the treatment of MM. Bortezomib-based regimens are now used as induction therapy before ASCT in transplant-eligible patients [6–9], and MP + thalidomide [10], MP + bortezomib [11], and lenalidomide + dexamethasone [12] are the widely used regimens for transplant-ineligible patients. Several clinical studies have shown an improvement of overall response rate and progression-free survival (PFS) in both transplant-eligible and transplant-ineligible patients by incorporating novel agents into antimyeloma therapy [13, 14]. However, the significant extension in overall survival (OS) has only been observed in younger patients under 60 years of age by population-based analyses (Table 1) [15–17]. Thus, the prognosis of elderly patients remains poor [18], and more effective strategies are needed to improve the outcome of those patients.

In the following section, we review the clinical trials of ASCT and discuss its role in the recent treatment strategies for elderly patients with MM.

## 2. Efficacy and Feasibility of ASCT in Elderly Patients

Aging is likely to be associated with reduction in organ functions and drug metabolisms. Accordingly, elderly patients aged 65 years or older are usually considered as ineligible for high-dose melphalan therapy (200 mg/m^2^) followed by ASCT. Thus, clinical trials of ASCT have been mostly undertaken in patients younger than 65 years of age, and reports of ASCT performed in elderly patients are hardly available.

The toxicity of each high-dose or intermediate-dose melphalan was evaluated in elderly patients by Badros et al. [19]. Four transplant-related deaths occurred among 25 (16%) patients aged 70 years or older with the melphalan dose of 200 mg/m^2^, but after reducing the dose to 140 mg/m^2^ transplant-related mortality was significantly reduced to 1 out of 45 (2%) patients while maintaining the efficacy. Palumbo et al. conducted a trial of 2-3 courses of ASCT with 100 mg/m^2^ of melphalan in 71 patients aged 55–75 years (median, 64 years old) without comorbidities [20]. When compared with the matched-pair patients treated with MP therapy, the efficacy of ASCT was significantly superior to the MP therapy in terms of complete response (CR) rate, event-free survival (EFS), and OS, without any transplant-related death.

Thereafter, elderly patients, if in a fit medical condition, have been considered to be eligible for ASCT irrespective of the chronological age in most institutions [21, 22]. Several single institution studies have reported the experience of ASCT in elderly patients as well as in younger patients (Table 2) [23–30]. As conditioning regimen before ASCT, reduced dose of melphalan to 100–140 mg/m^2^ has been used in the elderly patients. Notably, there was no significant difference in transplant-related mortality between elderly patients and younger patients. Recently, the transplant-related mortality has decreased to 3-4% probably due to the improvement of supportive therapies [31]. In terms of the efficacy, CR rate of the younger patients and that of elderly patients were 24–48% and 12–44%, respectively, and most studies have shown no significant difference in CR rates between the two age groups, younger and elderly. The median PFS for the younger patients and the elderly patients was 17–45 months and 17–29 months, respectively. Likewise, the median OS for the younger patients and the elderly patients ranged from 36 to 73 months and from 39 to 57 months, respectively, and there was no significant difference between the two age groups.

Taken together, reduced-intensity ASCT is considered to be a safe and effective therapeutic modality even in patients aged 65–75 years in a good performance status without comorbidities.

## 3. Efficacy of ASCT in Comparison with That of Chemotherapy

To compare the efficacy of ASCT with reduced-dose melphalan with that of conventional chemotherapy, several randomized controlled trials have been performed in elderly patients aged 65 years or older (Table 3).

Palumbo et al. conducted a randomized trial enrolling 194 patients to assess the efficacy of VAD induction therapy + tandem ASCT (100 mg/m^2^ of melphalan) versus MP therapy in patients aged 50–70 years [32]. Maintenance therapy with interferon + dexamethasone was provided for the responding patients in both treatment arms. In older patients aged 65 to 70 years, the median EFS was significantly extended in the ASCT group compared with the MP group (28.0 versus 16.4 months, resp., *P* = 0.023). The median OS was also significantly extended in the ASCT group compared with the MP group (58.0 versus 37.2 months, resp., *P* = 0.04). These results suggest that intermediate-dose melphalan would be an effective treatment approach in elderly patients aged 65 to 70 years.

Facon et al. conducted a randomized trial comparing MP therapy (*n* = 196), MP + thalidomide (MPT) therapy (*n* = 125), and VAD induction therapy + tandem ASCT (100 mg/m^2^ of melphalan, *n* = 126) in patients aged 65 to 75 years [33]. The median PFS was 17.8 months with MP, 27.5 months with MPT, and 19.4 months with ASCT, respectively, and there was no significant difference in PFS between the MP group and the ASCT group (*P* = 0.25). The median OS was 33.2 months with MP, 51.6 months with MPT, and 38.3 months with ASCT, and no difference was seen between the ASCT group and the MP group (*P* = 0.32). On the other hand, significantly longer PFS and OS were observed in the MPT group compared with the ASCT group (*P* = 0.0002 and *P* = 0.027, resp.). When compared with the results of Palumbo et al., the median OS with MP was similar, but the median OS with ASCT was shorter in the report of Facon et al. This was probably because maintenance therapy was not intended in the study of Facon et al.

Barlogie et al. reported that the median OS had reached to 60 months with the total therapy composed of induction therapy, tandem ASCT (140–200 mg/m^2^ of melphalan), and interferon + dexamethasone maintenance therapy in 136 patients aged 65 years or older [34]. Their results showed a marked improvement of OS in elderly patients, suggesting the importance of continuous treatment after ASCT.

## 4. New Treatment Strategy Incorporating Novel Agents and ASCT in the Elderly

Several recent clinical trials have been designed to evaluate the efficacy and feasibility of sequential treatment strategies, for example, induction with novel agents, consolidation with ASCT, and maintenance with novel agents. Palumbo et al. and Gay et al. conducted a phase II trial of induction therapy with 4 cycles of bortezomib + pegylated liposomal doxorubicin + dexamethasone followed by tandem ASCT (100 mg/m^2^ of melphalan), consolidation therapy with 4 cycles of lenalidomide + prednisone, and maintenance therapy with lenalidomide alone in patients aged 65–75 years (Table 3) [35, 36]. The median PFS was 48 months, and the 5-year estimate of survival was 63% among the total of 102 patients. In particular, the 5-year estimate of survival of the 54 patients who obtained a CR was excellent reaching to 83%. In terms of the safety profiles, three and five patients died during induction therapy and during ASCT, respectively. The treatment-related mortality was significantly higher in the older patients (5 of 26 (19%)) compared with that of the younger patients (3 of 76 (4%)) aged less than 70 years (*P* = 0.024). From these results, it would be concluded that a sequential approach including reduced-intensity ASCT may benefit patients younger than 70 years of age with good performance status and without comorbidities.

Straka et al. performed a randomized controlled trial of tandem ASCT (140 mg/m^2^ of melphalan) + lenalidomide maintenance therapy comparing with continued lenalidomide + dexamethasone therapy after induction therapy of 3 cycles of lenalidomide + dexamethasone and peripheral blood stem cell collection in patients aged 60–75 years [37]. Preliminary results have demonstrated a successful collection of peripheral blood stem cells in 97% of patients given induction therapy with lenalidomide + dexamethasone. By the long-term followup the importance of ASCT for elderly patients in the era of novel agents would be clarified.

## 5. Current Status of the Treatment in Elderly Patients

Thus, treatment strategy employing ASCT in patients aged 65–70 years has not been well established, and the indication of ASCT varies in each institution.

Kumar et al. have reported an improvement in OS of MM patients diagnosed during 2001–2006 period and ascribed it to the beneficial effects of novel agents [38]. More recently, they have updated the outcome of 1038 patients diagnosed and treated in Mayo Clinic between 2001 and 2010 [39]. Notably, when comparing the survival data between patients diagnosed during the period of 2001–2005 and that of 2006–2010, an improvement in survival was only observed in the older patients aged ≥65 years but not in the younger patients aged <65 years. A total of 393 patients (37%) received ASCT as initial treatment. When comparing patients receiving ASCT with those who did not receive ASCT, OS was similar when the analysis was confined to the younger age group, while it was significantly prolonged in the older age group (*P* < 0.01). Therefore, it is considered that the survival has markedly improved and has almost reached to its maximum in the younger patients; however, the survival of the elderly patients has just started to increase with the use of novel agents and an increasing application of ASCT to elderly patients.

Similarly, to assess the current treatment status, the Japanese Society of Myeloma has surveyed the outcome of patients aged 65–70 years including those enrolled in clinical trials as well as those in routine practice who had received an initial treatment between the years of 2004 and 2009 in collaboration with Dr. Palumbo and his colleagues of the Turin University of the European Myeloma Network [40]. The total number of the patients was 318 (268 from the Japanese Society of Myeloma and 50 from the Turin University) composed of 167 male and 151 female patients. M protein type was IgG in 169, IgA in 80, Bence-Jones protein (BJP) in 56, IgD in 7, and other types in 6. International staging system (ISS) stage was I in 86, II in 107, III in 102, and unknown in 23, respectively. As initial treatment, 192 patients were treated with conventional chemotherapy alone such as MP and VAD therapy, 88 with conventional chemotherapy + novel drugs such as bortezomib + dexamethasone and MP + bortezomib therapy, 21 with conventional chemotherapy (VAD) + ASCT, and 17 with novel drugs (bortezomib + dexamethasone) + ASCT, respectively. As a total, thirty-eight of the 318 patients (12%) aged 65–70 years were successfully treated with ASCT without any transplantation-related mortality.

**Figure 1:**
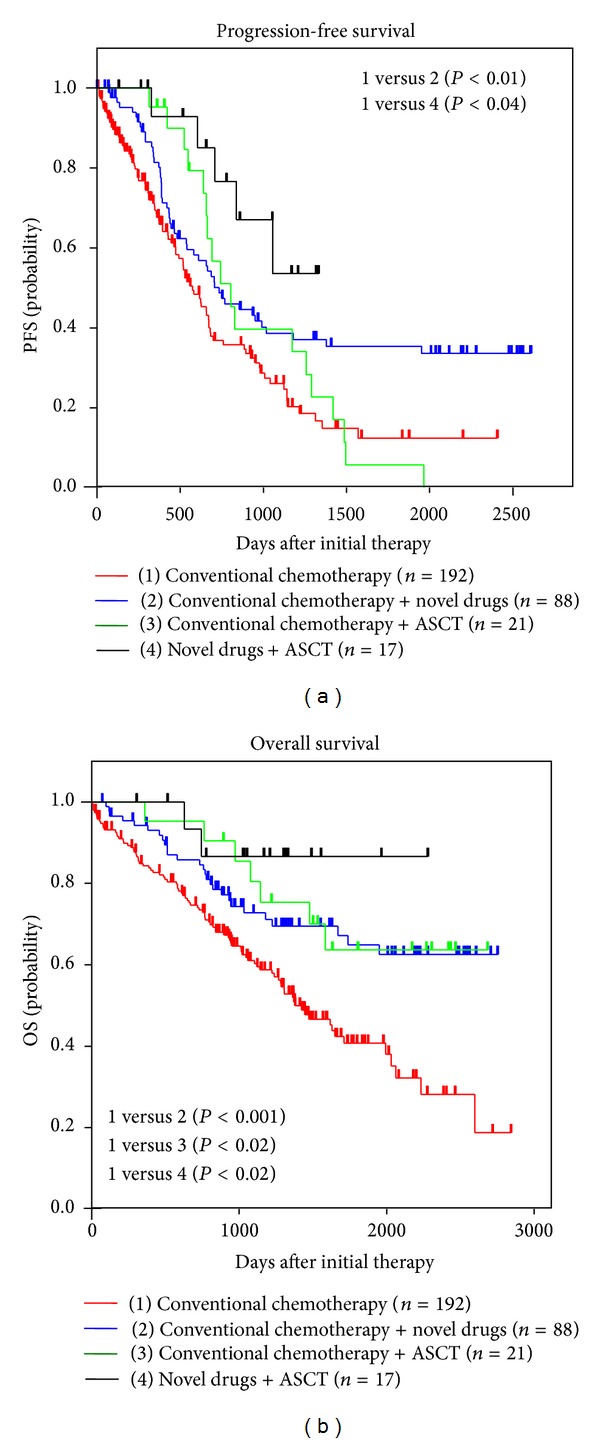
Retrospective analysis of outcome of newly diagnosed patients aged 65–70 years. Progression-free and overall survival according to the treatment groups such as conventional chemotherapy, conventional chemotherapy + novel drugs, conventional chemotherapy + ASCT, and novel drugs + ASCT is shown. Reproduced from [40] with permission from Karger.

The median PFS according to the different treatment modalities was 19.1 months for conventional chemotherapy group, 24.5 months for conventional chemotherapy + novel drug group, 26.8 months for conventional chemotherapy + ASCT group, and 35.2 months for novel drug + ASCT group, respectively, and PFS was significantly longer in the novel drug group (*P* < 0.01) as well as the novel drug + ASCT group (*P* < 0.04) in comparison with that of the conventional chemotherapy group (Figure 1), respectively. As for OS, the median OS of the conventional chemotherapy group was 46.0 months with other groups being not reached. Five-year estimates of survival were 40%, 62%, 63%, and 87%, respectively, and were significantly extended in the novel drug group (*P* < 0.001), the conventional chemotherapy + ASCT group (*P* < 0.02), and in the novel drug + ASCT group (*P* < 0.02) compared with the conventional chemotherapy group (Figure 1).

From this survey, it appeared that ASCT had been performed by the physician's discretion based on patient's condition irrespective of the age of 65 years or older. Although the patient characteristics of each treatment group vary, the clinical benefit represented by PFS and OS of the conventional chemotherapy + ASCT group was almost equal to that of the novel drug group. Furthermore, the induction therapy with novel drugs followed by ASCT consolidation seemed to have contributed to the significantly improved outcome. Therefore, ASCT can be an option even in the treatment of fit elderly patients [18].

## 6. Conclusion

ASCT can be applied to a selected patient of 65 years of age or older when performance status is fit and no comorbidities/complications are present. Recent studies have demonstrated that ASCT with intermediate-dose melphalan (100–140 mg/m^2^) is a safe and effective treatment modality in patients younger than 70 years of age. Although the benefit of reduced-intensity ASCT had not been clearly demonstrated in the past decade, it can be a viable option if incorporated into sequential treatment strategies along with novel agents. Therefore, the indication of ASCT should be seriously considered in each elderly patient based on the performance status and the presence of complications and/or comorbidities but not on chronological age alone. The sequential approach including ASCT can be challenging but would be feasible approach to further improve the outcome of elderly patients with MM.

## Figures and Tables

**Table 1:** Five-year estimates of relative survival in patients with multiple myeloma according to different age groups.

Authors (year)	Age group	Periods		*P* value
Brenner et al., (2008) [15]		1990–1992	2002–2004	
	50–59	38.8%	48.2%	0.001
	60–69	30.6%	36.3%	0.09
	70–79	27.1%	28.7%	0.21

Pulte et al., (2011) [16]		1998–2002	2003–2007	
	50–54	49.3%	58.3%	<0.05
	55–59	41.7%	52.5%	<0.05
	60–64	35.7%	44.4%	0.01
	65–74	32.1%	37.4%	<0.01
	≥75	19.4%	22.7%	0.06

Pozzi et al., (2013) [17]		1988–1996	2006–2009	
	<65	58.1%	74.2%	<0.001
	65–74	49.9%	72.9%	0.008
	≥75	29.2%	31.4%	0.567

**Table 2:** Comparison of clinical outcomes of ASCT according to different age groups.

Authors (year)	Median age (range)	Number of patients	Conditioning regimen	TRM	CR	Median PFS	Median OS
Siegel et al., (1999) [23]	52 (37–64)	49	MEL 200	2%	43%	34 mo	58 mo
	67 (65–76)	49		8%	20%*	18 mo	40 mo

Sirohi et al., (2000) [24]	55 (31–64)	17	MEL 200	12%	47%	23 mo	36 mo
	67 (65–74)	17		18%	35%	24 mo	43 mo

Reece et al., (2003) [25]	52 (30–59)	382	MEL ± TBI and others	6%	34%	27 mo	39 mo
	63 (60–73)	110		5%	33%	24 mo	39 mo

Jantunen et al., (2006) [26]	57 (39–64)	79	MEL 200	1%	36%	21 mo	66 mo
	68 (65–73)	22		0%	44%	23 mo	57 mo

Gertz et al., (2007) [27]	≤65	541	MEL 200	3%	30%	17 mo	44 mo
	>65	137	MEL 140–200	3%	40%	17 mo	44 mo

Kumar et al., (2008) [28]	56 (37–65)	60	MEL 200	0%	28%	18 mo	53 mo
	72 (70–76)	33	MEL 140–200	3%	42%	29 mo	NR

El Cheikh et al., (2011) [29]	62 (60–65)	104	MEL 140–200	4%	48%	45 mo	57% at 5 yr
	69 (65–77)	82	MEL 100–200	4%	41%	27 mo**	54% at 5 yr

Muta et al., (2013) [30]	60 (51–64)	63	MEL 180–200	3%	24%	21 mo	73 mo
	67 (65–76)	25	MEL 100–200	4%	12%	17 mo	41 mo

TRM: treatment-related mortality; CR: complete response; PFS: progression-free survival; OS: overall survival; MEL: melphalan; TBI: total body irradiation; mo: months; yr: years; NR: not reached.

*P = 0.02, **P < 0.0001.

**Table 3:** Results of the clinical trials of ASCT in elderly patients aged 65 years or older.

Authors (year)	Regimen	Number of patients	CR/nCR	≥PR	Median PFS/EFS	Median OS	TRM
Palumbo et al., (2004) [32]	MP	36	8%	50%	16.4 mo	37.2 mo	3%
	DAV + MEL 100	44	25%^∗1^	68%	28.0 mo^∗2^	58.0 mo^∗3^	7%

Facon et al., (2007) [33]	MP	196	2%	35%	17.8 mo	33.2 mo	2%
	MPT	125	13%^∗∗1^	76%^∗∗2^	27.5 mo^∗∗2^	51.6 mo^∗∗3^	0%
	VAD + MEL 100	126	18%^∗∗2^	65%^∗∗2^	19.4 mo	38.3 mo	5%

Gay et al., (2013) [36]	PAD + MEL 100 + LP-L	102	53%	95%	48 mo	63% at 5 yr	8%

CR: complete response; nCR: near complete response; PR: partial response; PFS: progression-free survival; EFS: event-free survival; OS: overall survival; TRM: treatment-related mortality; MP: melphalan + prednisone; DAV: dexamethasone + doxorubicin + vincristine; MEL: melphalan; MPT: melphalan + prednisone + thalidomide; VAD: vincristine + adriamycin + dexamethasone; PAD: bortezomib + pegylated liposomal doxorubicin + dexamethasone; LP: lenalidomide + prednisone; L: lenalidomide; mo: months, yr: years.

^∗1^ *P* = 0.05, ^∗2^ *P* = 0.023, ^∗3^ *P* = 0.04, ^∗∗1^ *P* = 0.0008, ^∗∗2^ *P* < 0.0001, ^∗∗3^ *P* = 0.0006 (in comparison with MP).
